# Identifying mechanisms for facilitating knowledge to action strategies targeting the built environment

**DOI:** 10.1186/s12889-016-3954-4

**Published:** 2017-01-03

**Authors:** Ghazal S. Fazli, Maria I. Creatore, Flora I. Matheson, Sara Guilcher, Vered Kaufman-Shriqui, Heather Manson, Ashley Johns, Gillian L. Booth

**Affiliations:** 1 Centre for Urban Health Solutions, The Keenan Research Centre, Li Ka Shing Knowledge Institute, St. Michael’s Hospital, 209 Victoria Street, M5B 1T8 Toronto, ON Canada; 2 Dalla Lana School of Public Health, University of Toronto, Toronto, ON Canada; 3 Leslie Dan Faculty of Pharmacy, University of Toronto, Toronto, ON Canada; 4 Institute of Health Policy, Management and Evaluation, University of Toronto, Toronto, ON Canada; 5 Institute for Clinical Evaluative Sciences, Toronto, ON Canada; 6 Department of Nutrition, Faculty of Health Sciences, Ariel University, Ariel, Israel; 7 Public Health Ontario, Toronto, ON Canada

**Keywords:** Built environment, Population health, Urban health, Planning, Transportation, Stakeholder engagement, Knowledge to action, Chronic diseases

## Abstract

**Background:**

In recent years, obesity-related diseases have been on the rise globally resulting in major challenges for health systems and society as a whole. Emerging research in population health suggests that interventions targeting the built environment may help reduce the burden of obesity and type 2 diabetes. However, translation of the evidence on the built environment into effective policy and planning changes requires engagement and collaboration between multiple sectors and government agencies for designing neighborhoods that are more conducive to healthy and active living. In this study, we identified knowledge gaps and other barriers to evidence-based decision-making and policy development related to the built environment; as well as the infrastructure, processes, and mechanisms needed to drive policy changes in this area.

**Methods:**

We conducted a qualitative thematic analysis of data collected through consultations with a broad group of stakeholders (*N* = 42) from Southern Ontario, Canada, within various sectors (public health, urban planning, and transportation) and levels of government (federal, provincial, and municipalities). Relevant themes were classified based on the specific phase of the knowledge-to-action cycle (research, translation, and implementation) in which they were most closely aligned.

**Results:**

We identified 5 themes including: 1) the need for policy-informed and actionable research (e.g. health economic analyses and policy evaluations); 2) impactful messaging that targets all relevant sectors to create the political will necessary to drive policy change; 3) common measures and tools to increase capacity for monitoring and surveillance of built environment changes; (4) intersectoral collaboration and alignment within and between levels of government to enable collective actions and provide mechanisms for sharing of resources and expertise, (5) aligning public and private sector priorities to generate public demand and support for community action; and, (6) solution-focused implementation of research that will be tailored to meet the needs of policymakers and planners. Additional research priorities and key policy and planning actions were also noted.

**Conclusion:**

Our research highlights the necessity of involving stakeholders in identifying inter-sectoral solutions to develop and translate actionable research on the built environment into effective policy and planning initiatives.

## Background

Over the last few decades, obesity rates have reached epidemic proportions globally, becoming one of the most pressing public health challenges of our time. Obesity is a major risk factor for type 2 diabetes, heart disease, cancer, and premature mortality [1, 2]. The economic impact of obesity is substantial, costing the Canadian economy $4.6 billion in 2008 [3]. Indirect costs are likely to be equally high due to development of chronic diseases, disability and premature loss of life [3]. While public health experts emphasize the need to decrease risk factors for obesity at a population level, rates of physical inactivity and unhealthy eating remain high in Canada [2].

In response to this public health challenge, policymakers and public health professionals have been searching for broad, policy-based solutions to help curb the rise in obesity [4, 5]. The notion that communities can be designed to promote healthy active living is an option that is gaining momentum [6–9]. A growing number of studies suggest that residents living in sprawling, car-dependent communities engage in far less walking or other forms of active transportation, and spend more time in cars compared to those living in older, more compact neighborhoods [7–17]. Furthermore, living in suburban neighborhoods is associated with higher rates of overweight and obesity, and a greater likelihood of developing diabetes [12, 18–21]. Therefore, interventions that support changes to the built environment through public policies and planning initiatives may help stimulate healthy lifestyle choices by providing opportunities for walking, cycling and other physical activities [22].

Despite the growing body of literature linking the built environment to health, little evidence has been translated to policy and planning actions [23, 24]. Designing or re-designing communities to optimize health is challenging as it requires collective, coordinated efforts of policymakers and planners across a range of sectors and organizations (i.e. public health, planning, transportation, economic development organizations, etc.) [4]. Thus, establishing efficient processes and mechanisms to promote cross-sectoral stakeholder engagement and collaboration will be needed to effectively implement such complex solutions.

The overarching goal of this work was to explore how evidence on the built environment could be translated into policy and planning actions to support healthy community design. More specifically, our objectives were to identify: (1) the infrastructure, processes, mechanisms and actions needed to drive such changes; and (2) current knowledge and information gaps and other barriers that impede policy and planning decisions related to the built environment.

## Methods

### Study design and setting

To address these objectives, we engaged key stakeholders who have an interest in built environment policies. This research focused on Southern Ontario, one of the largest and fastest growing regions in North America with a population of 8.76 million people in 2011, living primarily in urban centres [25]. The region is expected to grow an additional 3.7 million by 2031 and there has been a call for action to create more compact and complete communities to support healthy and active living [26].

### Participants

We recruited participants, including policymakers and planners, from a variety of sectors (public health, urban planning, and transportation) and levels of government (federal, provincial, and municipal). We used a purposive sampling strategy, [27] wherein public health representatives from major metropolitan areas in Southern Ontario (i.e. Toronto, Peel, Durham, York, Halton, Hamilton, London and Ottawa) who were known to the research team were asked to identify potential participants from regional planning and transportation departments (in each metropolitan area) with whom they were working. We also identified key representatives from different Ministries within the Ontario government, non-profit professional organizations, and peer-review granting agencies – to broaden the spectrum of stakeholders that could provide insight on both existing and upcoming policies as well as research funding initiatives related to the build environment. These stakeholders were invited to participate in a full-day stakeholder engagement meeting. Our recruitment strategy resulted in a diverse sample of participants (*N* = 42) from government and non-government agencies such as planning (*n* = 12), transportation (*n* = 10) and public health (*n* = 20).

### Data collection

Participants attended a full-day stakeholder engagement meeting in October 2013. Prior to the meeting, we sent participants an open-ended survey questionnaire to gather information on existing research priorities and initiatives in their regions to determine their level of engagement and readiness to support actions related to the built environment. Results from the pre-meeting survey formed the basis for discussions with stakeholders during the engagement meeting. During the day, we held facilitated small group discussions. The participants were asked to identify solutions across different sectors to transform evidence on the built environment into policy and planning initiatives, as well as research priorities and key actions that might be needed to achieve these goals. Specifically, we asked participants to identify: a) information and tools needed to advance healthy built environment initiatives in their local regions; b) knowledge transfer activities that would enable research findings to have a meaningful impact on policy and planning changes; and c) implementation strategies to help move this knowledge from evidence to action. To facilitate discussion, participants were grouped initially by sector (e.g. planning, public health, and transportation) and subsequently by region. Two note-takers were assigned to transcribe participant discussions using laptops, and these data were later compiled and analyzed using qualitative thematic analysis, as described below.

### Qualitative thematic analysis

Data from stakeholder consultations were analyzed through qualitative thematic analysis by adopting an inductive approach that would allow for themes to emerge from the transcripts without any preconceived topics or directions [27]. Transcripts were first reviewed independently by two members of the research team (GF and MC) to generate an initial list of themes. Three additional researchers (SG, VKS, GB) then independently analyzed the same transcripts from the stakeholder engagement meeting to confirm the initial list of themes and to identify any additional themes. The final list of themes were discussed among team members and assessed for saturation of ideas as related to the research objectives. In addition, two members (SG, VKS) of the research team independently re-analyzed the data to identify research priorities and key next steps to address the challenges and opportunities for translating evidence on the built environment into policy and planning decisions. Overall, there was little disagreement in the final list of themes, research priorities and next steps; any discrepancies were discussed among all team members to achieve consensus.

A post-event survey was conducted via email to gain feedback from participants on the importance of each theme identified in the qualitative thematic analysis process, by rating it on a scale of 1 (not important at all) to 5 (very important). We asked participants to align each theme with potential policies or strategies that would positively enhance the built environment in Southern Ontario. On average, all the themes were rated as important or very important. Using a likert scale, participants also ranked each research priority and knowledge gap and key policy and planning action on the built environment. We calculated the average ranking of each research priority/ knowledge gap and key policy and planning action item. The rankings were divided into top and lower priorities.

To aid in the interpretation and discussion of results, we used an adapted version of the Knowledge to Action (K2A) framework created by the Centre for Disease Control and Prevention (CDC) [28], to classify themes to one of the three phases (research, translation, and implementation) required to develop and translate actionable research into effective policies targeting the built environment [28].

## Results

### Overarching themes

The analysis revealed five themes on knowledge gaps, barriers and challenges in translating evidence on the built environment and health into policy and planning decisions, and implementation strategies needed to do so. A detailed description of each theme is provided below and shown in Table 1, where the CDC K2A framework is cross-referenced to reflect the alignment between each identified theme and the K2A cycle [28].

**Table 1:** Themes from stakeholder consultations on infrastructure, processes, and mechanisms that will facilitate positive modifications to the built environment

Themes	Knowledge to Action Cycle
Policy informed and actionable research	
• Health economic evaluations for estimating costs, benefits and impacts • Evaluation of natural policy experiments locally and abroad • Examine effects among priority populations (what works for whom and under what context)	Knowledge Generation
Targeted and Impactful Messaging	
* •* Build multimedia strategy to communicate evidence across sectors * •* Tailor the message for different audiences (politicians vs. public) * •* Powerful messaging through infographics, maps, fact sheet	Knowledge Translation
Common Measures and Tools	
• Make user-friendly data on the built environment available for policymakers and planners	Knowledge Generation
• Improve co-ordination & alignment of methods for measuring the built environment	Knowledge Translation
• Develop and implement standardized metrics and performance measures to enhance capacity for ongoing monitoring, reporting, and surveillance	Implementation
Intersectoral collaboration and alignment within and between levels of Government	
• Align agendas and find common goals • Identify political “Champions” • Seek financial support for cross-sectoral interventions and evaluations • Develop mechanisms for cross-sectoral performance measurement (benefits and impacts)	Implementation
Importance of Public and Private Sector Advocacy	
• Seek support from NGOs and private industries • Identify community ‘Champions’ and ‘Brokers’ • Roll out social marketing campaigns • Develop mechanisms to coordinate advocacy efforts of different groups	Implementation
Solution-focused implementation	
• Account for political context in all activities (identify important policy milestones and “critical windows” for policy change • Support local initiatives through provincial legislation • Create tools to evaluate interventions for effectiveness & impact	Implementation

### Policy informed and actionable research

The need for policy-informed and actionable research was a recurrent theme among participants across various sectors and levels of government. Stakeholders identified four research priorities: 1) understanding the collective benefits of built environment changes on the health and wellbeing of the population; 2) identifying threshold effects of built environment attributes and their health impact on different populations (“*what works, for whom, and in what context*”); 3) assessing the health economic impact (costs and benefits) of competing policy options; and 4) evaluating the impact of natural policy experiments targeting the built environment. Research evidence that considers these approaches is needed in order to build a *business case* for changes in policies targeting the built environment. As several participants pointed out, we *“maybe need to create a business case for [the] physical environment”, “built environment changes must work in the context of economics…health priorities are understood, but we need to compare costs and benefits of changes to the built environment”.* As further noted “*economics is an issue because the payoff is very long-term for built environment changes*”; however “*if there is evidence of an impact on health it may provide evidence for the true need of action even if it costs more*”.

### Targeted and impactful messaging

Participants identified effective and timely communication of research findings to policymakers, planners, and the public as an important step to guide the adoption of evidence on the built environment into policy and practice. For research to have the greatest impact, it needs to be accompanied by tailored, impactful, and solution-oriented messages that reach all target populations and sectors. In other words, messaging needs to increase awareness and engender buy-in from politicians and the public-at-large as reflected in this quote from a participant, “*Helpful to have a ‘simple sell’ – a simple and clear message from the research”; although many cautioned that messages need to be “tailored to different knowledge users,* including *the public, policymakers, media, planners, and engineers”.* Participants suggested engaging in “*public consultation” to promote awareness, “prompt grass roots movements”*; and to provide an avenue for which public input can then be fed back to inform policies and research. Politicians were noted to be a key target for knowledge translation efforts to champion active transportation as a major issue. This is a vital step for creating the political will to develop policies and planning initiatives to promote healthy community designs.

### Common measures and tools

Participants unanimously identified a lack of user-friendly and available data for municipal public health and planning departments, and other agencies as a common challenge. While studies evaluating the built environment have grown rapidly over the past decade [10, 11, 19–24], gaps in data acquisition and consistent methods for measuring built environment characteristics have hampered the adoption of evidence into planning and practice. Participants suggested “*better co-ordination, alignment, and standardization of built environment metrics and performance measures”* that could be “*easily adopted by policymakers and planners*”; and greater access to low cost or publicly available data sources (including “*open access data*”) that would increase the capacity for ongoing surveillance work, allowing for comparisons over time and across regions. There was a desire too for more “*granular*”, “*localized data*” to contextualize “*local needs*” in order to “*tailor changes to individual municipalities*“, rather than taking a “*one-size-fits-all approach*” towards how evidence of the built environment is implemented into policy.

### Inter-sectoral collaboration and alignment within and between levels of government

The need for collective actions was emphasized by all participants regardless of their sector. As noted by one participant, “(*We) need more collaboration between silos…and some setup/communication infrastructure that allows sectors and governments to talk to each other”*. Furthermore, there needs to be “*horizontal accountability – bringing together people from different areas*” and “*a convergence of local and provincial government leaders to get things done*”. As an example, higher levels of government could create the legislative environment and provide the necessary resources (e.g. funding, tools, and data platforms) to support local policies at the municipal level. Participants mentioned that collaboration could be accomplished by: aligning agendas to find common goals across different sectors (planning, public health and transportation) and levels of government (federal, provincial, and municipal); identifying “political *champions”* to bring the built environment evidence to the forefront of policymaking –for example, “*real political leadership to push forward our evidence and priorities*”, *perhaps through a “Chief Planner counterpart to the Medical Officer of Health”*. Additional priorities include seeking financial support for implementation research; and developing mechanisms for performance measurement to understand the impact of healthier communities on population health –as well as its broader “*environmental and social benefits*”. To support cross-sectoral and cross-regional collaborations, some suggested developing the infrastructure for sharing expertise and experiences on built environment interventions on a broad scale, in a way that builds on existing networks. This could provide a unique “*data repository*” that would offer a “*best practices portal*” and facilitate the design and evaluation of natural policy experiments. Several options were put forth regarding where the governance and accountability of such an initiative would lie – with one participant recommending a “*national collaborative center for environment health*” to oversee this infrastructure to provide oversight and sustainability.

### Aligning public and private sector priorities

Participants also suggested the “*need to modify perceptions of both public and private sectors to generate public demand and support for community action*”. Strategies to address this issue may include: “*seeking support from non-government organizations and private industries to understand their priorities in implementing changes to the built environment”; “identifying community ‘champions’* to act as *‘brokers’ or agents of change in this process”; “developing social marketing campaigns”*; and other mechanisms to coordinate advocacy efforts of different organizations working in the public and private sectors in order to collaborate in a more cohesive way and achieve common goals. It was noted that “*behavioral economics may have useful insights/ideas for encouraging “better” behaviors”;* an example being an “*insurance company cutting their premiums for less miles driven”.* However, several participants highlighted the need to change social norms, noting, for example “*people think it’s safer to drive their kids to school, although statistically it’s not*” – and the difficulties in doing so; querying “*how do you drive culture change?*”. Participants identified the need for “*some sort of social marketing campaign around urban design*” that would link “*active transportation to weight loss*” akin to messaging campaigns encouraging stair use.

### Solution-focused implementation

An important contribution of implementation research is that it promotes a better understanding of how to effectively and efficiently translate evidence into action by considering the political contexts in all activities. Participants highlighted the importance of bringing researchers and policymakers together to tailor the research to be more solution-focused and to meet the needs of policymakers and planners. *“An important contribution of implementation research is that to increase walkability and transit options, we need to get regional organizations like the province on board with the local issues. There needs to be convergence of local and provincial government leaders to get things done. So, to do solution-focused implementation how do you get politicians on board? We all have our own agenda but we’re not all going to get what we want. If there are competing agendas, decisions often depend on the politics of the day”.* In order for policies and programs to have an optimal impact, they need to capitalize on “*critical windows for initiating change*”– for example, when existing policies are up for renewal – and account for local political and economic contexts of the region.

### Research priorities and knowledge gaps

**Fig. 1:**
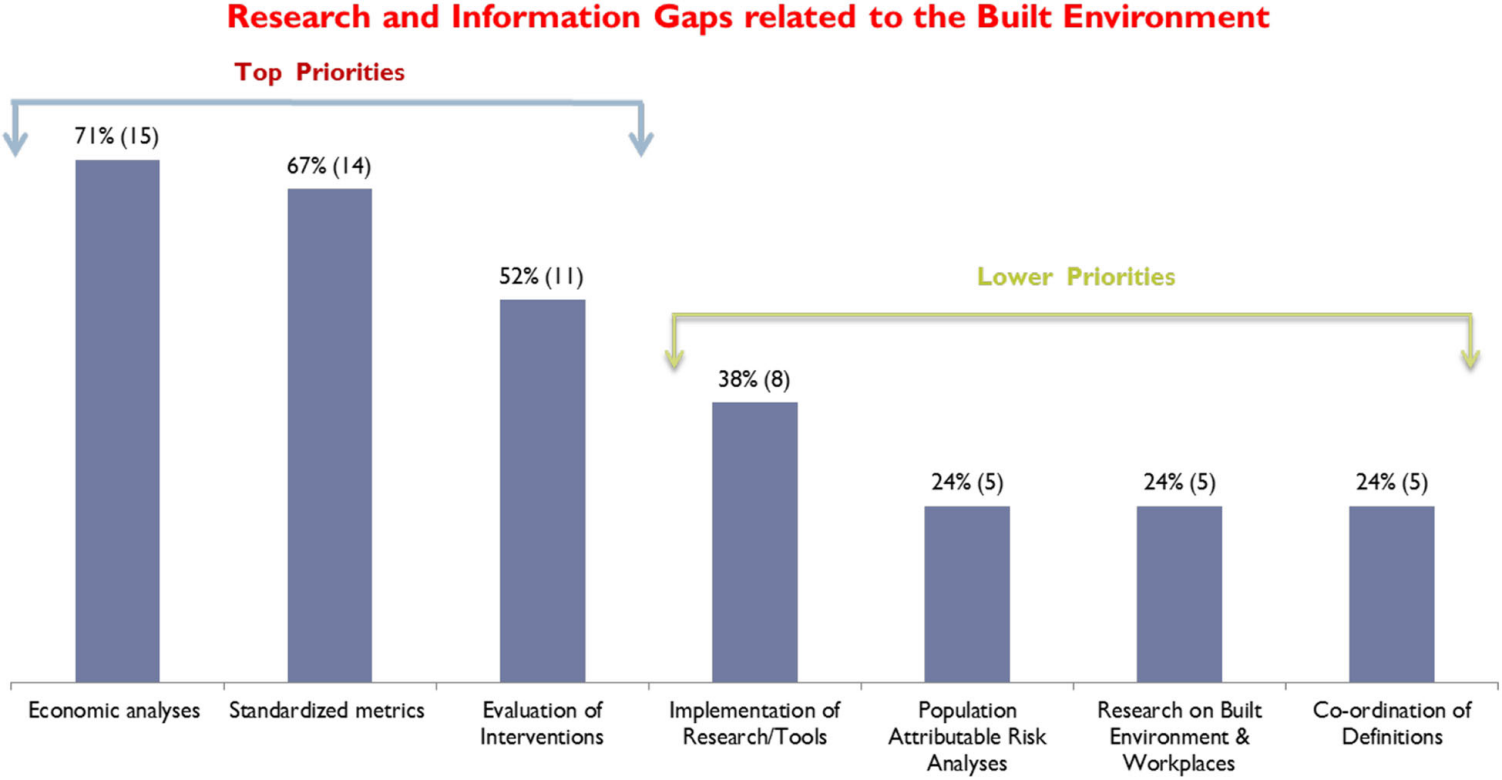
Research and information gaps with respect to *evidence* that can help promote actions and policies on the built environment, ranked as *top* and *lower* priorities by stakeholders

Participants provided important insights on actionable research priorities and opportunities as next steps that would address current knowledge gaps and facilitate the effective application of evidence on the built environment into policy and planning initiatives. These research and knowledge gaps are shown as top or lower priorities in Fig. 1 and reflect potential barriers that may impede policy and planning actions to improve the built environment. Some of the top research priorities included economic analyses on the benefits of policy and planning changes made to the built environment and standardized metrics and methodologies for measuring the built environment. Examples of items that were rated less highly included research approaches that may be of greater interest to scientists than politicians (e.g. population attributable risk analyses) and research on built environment and workplaces.

### Policy and planning actions on the built environment

**Fig. 2:**
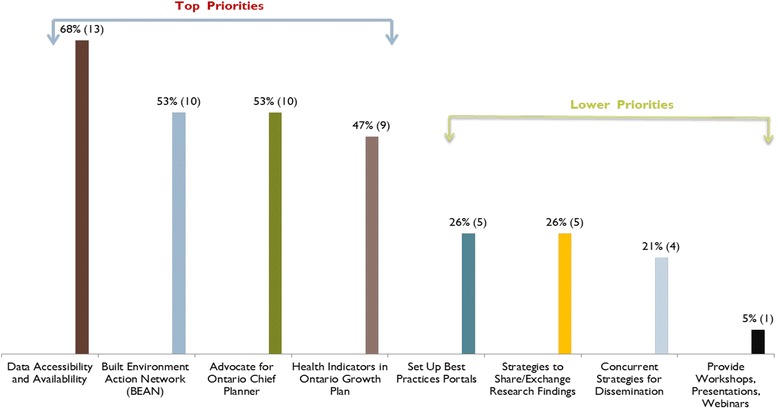
Summary of recommended next steps to support *policy and planning* actions related to the built environment

Key policy and planning actions recommended by participants that would support changes related to the built environment are provided in Fig. 2 (in order of priority) based on stakeholder input and ranking. For example, actions that were rated most highly included establishing a Built Environment Action Network (BEAN) for sharing data, resources and other tools, creating a leadership position in the Ontario Government for a Chief Planner, and developing health indicators as part of the evaluation of Ontario’s Official Growth Plan.

## Discussion

Identifying policy solutions to reduce the burden of obesity and related morbidity is an important call for action in public health. We engaged a broad group of stakeholders to share their thoughts and insights on key strategies for moving evidence on the built environment into action. While there was overall enthusiasm among policymakers, planners and advocacy groups to create healthy and active communities, systemic barriers to translating evidence into effective policy and planning initiatives need to be addressed in order to do so. In this study, we discovered several important themes, research opportunities and key policy and planning steps to help overcome the systemic barriers identified by participants for translating evidence on the built environment.

One major knowledge gap identified by our participants was the lack of cost-benefit analyses to support an economic argument for policy changes in how communities are designed. Furthermore, if neighborhoods that are designed to promote physical activity have lasting benefits on the health of the population by reducing obesity and related illnesses, then decision-makers will need to adopt a broader societal perspective in developing policies within their own area since investments borne by non-health sectors (e.g. municipal development, transportation and planning initiatives) may be cost-beneficial for the health system [29]. Changing the built environment is a major undertaking that requires vast inter-sectoral and financial investments in order to create the necessary infrastructure that supports healthy and active lifestyles [4]. In recent years, there have been major movements towards public transit expansion throughout the Greater Toronto Hamilton Area by planning, designing and building a more connected and integrated transportation network [30]. Official plans for municipalities can consider how best to support changes in transportation and development standards for building safe, healthy and sustainable communities. In this study, participants felt that one strategy to support ongoing evaluation would be to develop health indicators that can then be linked to the province’s official plan for community development. Hence, inter-sectoral collaborations and alignment between government levels and other sectors –important themes from this study – are necessary not only to drive change but also to assess the impact that such investments have had from a broad, societal perspective.

Several North American jurisdictions have developed policies to facilitate changes to the built environment. As part of a collaborative initiative in 2011, the Region of Peel in Ontario developed an evidence-based implementation tool called the Healthy Development Index (HDI) to aid in the evaluation of development initiatives submitted to the region and to encourage land use planning in a way that promotes active transportation [31]. The HDI is a scientifically validated tool that sets specific requirements for development projects, including new engineering standards and a requirement for a health background study prior to submission [31]. Development of such a tool requires significant political will and alignment of political agendas across multiple sectors and levels of government. Furthermore, the World Health Organization developed the Health Economic Assessment Tool (HEAT) to support health impact assessments of future planning and development of infrastructure [32]. A variety of professionals across different sectors can now use HEAT to evaluate the economic value of health benefits associated with physical activity by walking and cycling [32]. Similarly, the Integrated Transport and Health Impacts Model (ITHIM) developed in Cambridge, United Kingdom, is another evidence-based tool also designed and validated to estimate the health and environmental impacts of walking and cycling (or travel-related physical activity) [33]. Participants in our study supported the notion that policy informed and actionable research will need to consider the costs and benefits of built environment interventions in order to compare cost savings to health systems with investments in infrastructure planning and transportation. While validated tools that set criteria for health impact assessments are necessary, there is also a need for garnering federal support to form national collaborative coalitions or action networks that can bridge the gap between evidence, policy and planning related to the built environment. For example, Smart Growth America is a national coalition sponsored by the U.S. Department of Transportation, U.S. Department of Housing and Urban Development and the U.S. Environmental Protection Agency, which has resulted in numerous initiatives to create active communities [34]. Many regions in the U.S. including Rockville, Maryland, Denver, Colorado and San Diego, California have adopted Smart Growth principles to build more compact, walkable and mixed-used communities with access to local parks and public transportation [34]. However, a ‘culture of physical activity’ is a vital ingredient, as demonstrated in several European cities. For instance, Amsterdam and Copenhagen are recognized globally as world leaders in cycling networks [35]. Copenhagen has 400 km of bicycle lanes and over 36% of city residents travelling by bicycles, [35] supporting the notion that designing neighborhoods and streets that are conducive to active living can indeed promote physical activity. While there is growing evidence globally that initiatives designed to modify the built environment can encourage healthier behaviors, our findings, supported by other research [1, 4], indicate that to improve the built environment major political buy-in and support are necessary from the federal and provincial (or state) governments to ensure sufficient resources and effective collaborations –both horizontally (across sectors) and vertically (across government agencies) to enact change.

In moving built environment initiatives forward, participants in our study identified the importance of creating a Built Environment Action Network to develop and sustain synergistic partnerships between research, policy, planning and advocacy efforts. Additionally, this network could serve as a platform for sharing common tools and metrics for surveillance purposes and mechanisms for developing and implementing policy changes, as well as successes, challenges and failures in efforts to do so. Additionally, creating a new position within the province for a Chief Planner (akin to the role of Chief Medical Officer of Health), could provide the leadership and momentum to create the necessary cross-sectoral partnerships –within and across levels of the government for achieving immediate (i.e. zoning bylaws) and long-term (i.e. legislation) goals to improving the built environment. These recommendations can be supported by better integration and engagement of research and policy to understand the knowledge gaps and barriers that need to be overcome before policy decisions are made. Furthermore, the knowledge-to-action cycle can support the process of generating evidence, exchanging and disseminating information and evaluating the performance of policies and planning changes targeting the built environment. The impact of such natural policy experiments can then be shared and applied across regions.

This study has several limitations. First, these data were drawn from group discussions during a full-day in-person meeting as opposed to one-on-one interviews; therefore, the beliefs and perceptions of all the participants may not have been fully heard. Also, while two note-takers per group were present to record the discussions, some points may have been missed or incompletely captured. However, individuals participated in discussions that were grouped in two distinct ways – by sector and by region, which increased the opportunity for providing individual input. One of the key strengths of this research was the ability to incorporate insights and perspectives of multiple sectors and agencies who are actively involved in either generating or using evidence on the built environment. However, the data generated were based on stakeholders in one region of the country - urban centers in Southern Ontario. Therefore, our findings may not be fully generalizable to other jurisdictions. Nevertheless, prior research suggests that the themes we identified are relevant to other Canadian cities [1]. Furthermore, findings from this study (i.e. Built Environment Action Networks or BEAN, creating an Ontario Chief Planner position, and adding health indicators to the Ontario Growth Plan) are unique and may apply to other jurisdictions that are also likely grappling with the challenges of overcoming barriers in translating the evidence on the built environment into policy and planning. Finally, it was beyond the scope of the study to engage end-users such as community residents for input on ways to improve the built environment. Given that participants identified a need to engage community champions this should be an important consideration in built environment initiatives.

## Conclusion

Research shows that the environment in which we live has implications for our overall health and well-being [7–13]. This study identified several important themes related to the infrastructure, processes and mechanisms necessary to facilitate positive changes to the built environment. Study participants identified a series of actions and opportunities for public health, planning and transportation sectors and government agencies to consider for future planning and decision making in building neighborhoods and cities. Interventions targeting the built environment will require concerted efforts from champions across many policy and planning spheres and collaboration of stakeholders across public, community and private sectors to effectively apply evidence on the built environment. Understanding how to achieve changes in built environment to support healthy active living and reduce the burden of obesity and related illnesses is an important call for action on investments in population health and identifying system-wide barriers and knowledge gaps is an integral step to facilitating future research partnerships, and actions needed to drive such changes.

## Abbreviations

BEANs: Built Environment Action Networks
K2A: Knowledge to Action framework created by the Centre for Disease Control and Prevention (CDC)
